# Player detection method based on scale attention and scale equalization algorithm

**DOI:** 10.3389/fnbot.2023.1289203

**Published:** 2023-12-06

**Authors:** Pan Zhang, Jiangtao Luo

**Affiliations:** ^1^School of Communication and Information Engineering, Chongqing University of Posts and Telecommunications, Chongqing, China; ^2^Data Recovery Key Laboratory of Sichuan Province, Neijiang Normal University, Neijiang, China; ^3^Electronic Information and Networking Research Institute, Chongqing University of Posts and Telecommunications, Chongqing, China

**Keywords:** multi-scale target detection, scale attention, SIoU, scale equalization, implicit feature fusion

## Abstract

**Introduction:**

Object detection methods for team ball games players often struggle due to their reliance on dataset scale statistics, resulting in missed detections for players with smaller bounding boxes and reduced accuracy for larger bounding boxes.

**Methods:**

This study introduces a two-fold approach to address these challenges. Firstly, a novel multi-scale attention mechanism is proposed, aiming to reduce reliance on scale statistics by utilizing a specially created SIoU (Similar to Intersection over Union) label that explicitly represents multi-scale features. This label guides the training of multi-scale attention network modules at two granularity levels. Secondly, an integrated scale equalization algorithm within SIoU labels enhances the detection ability of multi-scale targets in imbalanced samples.

**Results and discussion:**

Comparative experiments conducted on basketball, volleyball, and ice hockey datasets validate the proposed method. The relative optimal approach demonstrated improvements in the detection accuracy of players with smaller and larger scale bounding boxes by 11%, 7%, 15%, 8%, 9%, and 4%, respectively.

## 1 Introduction

In team sports, such as basketball, volleyball, and ice hockey, the precise detection of players serves as the fundamental basis for intelligent auxiliary analysis of player movement data, assessment of multi-player coordinated behaviors, and comprehensive team technical and tactical analysis (Lu et al., 2011, 2013; Nishikawa et al., 2017; Stein et al., 2018; Kong et al., 2020). However, in the aforementioned competition scenarios, the statistical distribution of players' bounding boxes becomes wider and unbalanced due to the diversity of shooting distances and angles, along with the continuous movement and random switching of the camera. Specially, this substantial imbalance impairs the detection and localization abilities of existing model algorithms, particularly concerning extremely small and extremely large scale bounding boxes targets. Therefore, enhancing the detection ability of multiple players in non-equilibrium scale statistical scenes has become a significant challenge in the research and improvement of numerous algorithms in the field of computer vision.

As for the improvement of traditional algorithms, the primary emphasis lies on explicit multi-scale feature acquisition and fusion. In Lu et al. (2011), the combination of Histogram of Oriented Gradients (HOG) with color information is proposed. Stein et al. (2018) suggests the fusion of color histograms with target center points. Additionally, Santhosh and Kaarthick (2019) introduces the combination of the Deformable Parts Model (DPM) with Scale Invariant Feature Transform (SIFT) keypoints. These methods can significantly enhance the ability to extract explicit features of players through artificially designed operators. However, they exhibit more localized effectiveness and encounter difficulties in adaptively detecting targets of all scale bounding boxes.

The improvement based on deep learning models primarily leverages the universal object detection framework and its extensions (Akan and Varli, 2022; Sah and Direkoglu, 2023) to achieve the acquisition and fusion of implicit multi-scale features. As demonstrated in Nishikawa et al. (2017), the multi-branch output structure of the enhanced YOLOv3 model is directly employed to acquire and merge adjacent scale basketball player features. Building upon the addition of various scale feature detection branches, Kong et al. (2019) further integrates a spatial pyramid pooling (SPP) module, enhanced by hole convolution, into the training of the medium scale detection branch with the largest sample volume. This integration aims to enhance the complexity and precision of feature extraction and mitigate potential model overfitting or underfitting arising from sample imbalance. In Buric et al. (2019), features from non-adjacent scales were fused by integrating improved Feature Pyramid Networks (FPNs) into the backbone network, and the Fast R-CNN model was combined to enhance the detection effectiveness of multi-scale football players. Simultaneously, incorporating an attention mechanism into the backbone network for multi-scale feature extraction and fusion is also a prevalent approach. In line with this, both Komorowski et al. (2020) and Hurault et al. (2020) utilize attention mechanisms to enhance the detection capability of football players. In He (2022), attention mechanism was combined with a encoder-decoder model to obtain and fuse multi-scale features through encoding and decoding, achieving the detection of multiple types of multi-scale players. However, the naturally formed player detection dataset still exhibits an imbalance in the distribution of scales, resulting in a significant number of omissions in the detection of players with small sacle bounding boxes and inaccurate positioning of players with large scale bounding boxes in the aforementioned improved algorithms.

In response to the above issues, and inspired by techniques from partial feature fusion (Zhang et al., 2022) and data processing (Ding et al., 2023), this article proposes a multi-scale attention mechanism that weakly relies on the scale statistical distribution features of the dataset and a scale equalization algorithm. These methods combine the strong implicit feature extraction ability of deep learning models with the local enhancement characteristics of traditional operators describing explicit features, thereby further improving the accuracy of multi-scale player detection. The main innovations and contributions of this article include: (1) The proposal introduces the Similar to Intersection over Union (SIoU) label to represent explicit feature information of multi-scale targets. Based on this label, relevant network modules are constructed to generate coarse-grained scale attention feature planes that aid in multi-scale target detection. (2) An algorithm combining non Supervised learning and interval estimation using the statistical distribution information of the coarse-grained scale attention feature plane is proposed, so as to form a fine-grained scale attention with higher concentration. (3) We presents a scale equalization algorithm that is attached to the SIoU label and integrated into the training of the scale attention generation module. The algorithm aims to address the issue of network overfitting during training, which arises from the presence of a significant volume of samples with identical scale targets. Additionally, it mitigates the training error caused by the imbalance in the scale distribution of players' bounding boxes in ball team competitions.

## 2 The principle of SIoU label

**Figure 1:**
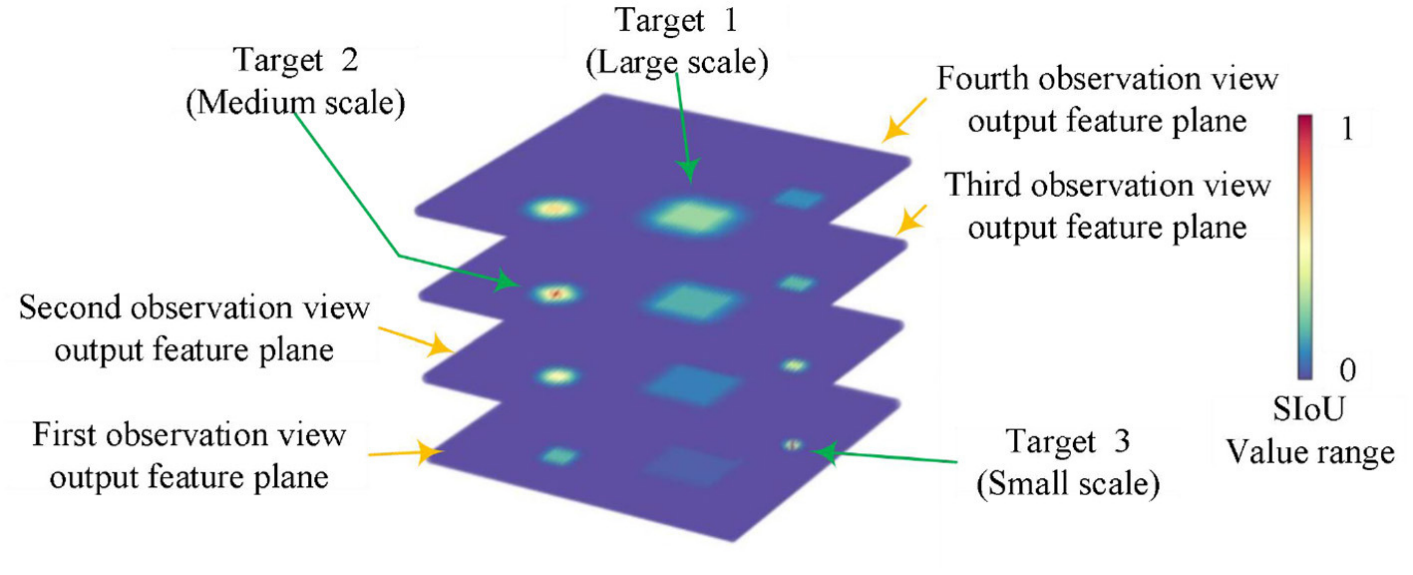
The visualization of SIoU features across distinct scales for various targets.

**Figure 2:**
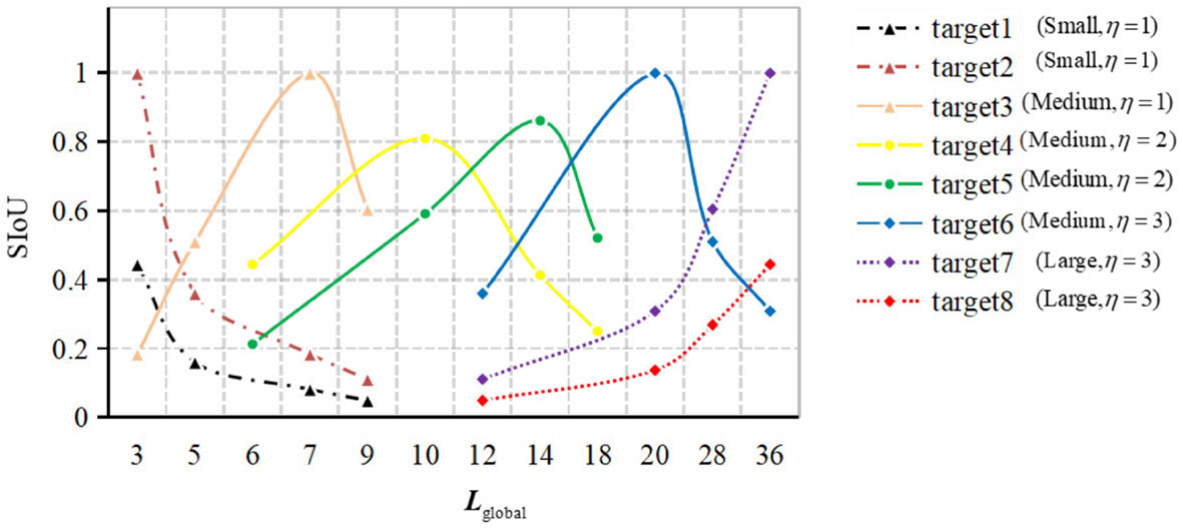
Numerical distribution of SIoU values for typical scale targets.

The Intersection over Union (IoU) (Yu et al., 2016) is a metric commonly employed in object detection tasks to assess algorithm performance. It is defined as the ratio between the intersection and union of the predicted field of view bounding box and the target's actual bounding box. This article formulates equation (2) using equation (1) to compute the SIoU (Similar to Intersection over Union) label. The SIoU label represents the ratio of intersection and union between the predicted field of view bounding box and the actual bounding box of the target in the output feature plane of the observed field of view. It calculates this value while continuously shifting the center position (*x, y*) of the predicted field of view bounding box. *S*_target_(*k*) denotes the true bounding box of the k-th target, and *S*_kernel_(*x, y, z*) represents the predicted bounding box of the z-th observation field when the output feature plane is centered at point (*x, y*). The SIoU values that can be generated through systematic variation of the size of the predicted field of view bounding box and the target's actual bounding box are illustrated in Figures 1, 2. This numerical characteristic of change exhibits similarity to the credibility of the human visual system when observing multi-scale targets across different fields of view, thus providing an explicit expression of multi-scale characteristics.


(1) $$\begin{array}{l} \text{IoU}=\frac{S_{\text{overlap}}}{S_{\text{union}}} \end{array}$$

(2) $$\begin{array}{l} \text{SIoU(}x\text{,}y\text{,}z\text{,}k\text{)}=\frac{S_{\text{overlap}}\text{(}x\text{,}y\text{,}z\text{,}k\text{)}}{S_{\text{union}}\text{(}x\text{,}y\text{,}z\text{,}k\text{)}}=\frac{S_{\text{target}}\text{(}k\text{)}\cap S_{\text{kernel}}\text{(}x\text{,}y\text{,}z\text{)}}{S_{\text{target}}\text{(}k\text{)}\cup S_{\text{kernel}}\text{(}x\text{,}y\text{,}z\text{)}} \end{array}$$

Figure 2 displays a representative statistical distribution of SIoU values, obtained through a typical single point quantization calculation, applied to targets of various scales using four corresponding equivalent prediction field of view boundary boxes. The typical single point quantization value refers to the SIoU value calculated when the predicted field of view bounding box aligns precisely with the center position of the target's actual bounding box. This serves as an illustrative example of certain feature points in Figure 1.

In Figure 2, the distinct line types represent different predicted branches η to which the target belongs. The calculation of these branches is determined by equation (3), where η_max_ denotes the upper limit of the number of predicted branches in the model. In equation (3), ℓ_target_(*k*)denotes the edge length of the k-th target, which is computed following equation (4). Likewise, $\ell_{\text{kernel}}^{z}\left(x_{\text{center}},y_{\text{center}}\right)$ signifies the edge length of the z-th basic predicted field of view bounding box, calculated based on equation (5). The set *L*_global_, comprising the edge lengths of all globally equivalent predicted field of view bounding boxes in the figure, is derived following equation (6).


(3) $$\begin{array}{l} \eta =\min\left(\max\left(\log_{2}^{\frac{\ell_{\text{t}\arg\text{et}}\left(k\right)}{\max\left(\;\left\{\;\ell_{\text{kernel}}^{z}\text{(}x_{\text{center}},y_{\text{center}}\text{)}\right\}\;\right)}}+2,1\right),\eta_{\max}\right) \end{array}$$

(4) $$\begin{array}{l} \ell_{\text{t}\arg\text{et}}\left(k\right)=\sqrt{S_{\text{target}}\left(k\right)} \end{array}$$

(5) $$\begin{array}{l} \ell_{\text{kernel}}^{z}\text{(}x_{\text{center}},y_{\text{center}}\text{)}=\sqrt{S_{\text{kernel}}\text{(}x_{\text{center}},y_{\text{center}},z\text{)}} \end{array}$$

(6) $$\begin{array}{l} L_{global}=\left\{\ell_{\mathrm{kernel}}^{z}\left(x_{\mathrm{center}},y_{\mathrm{center}}\right)\times 2^{\eta -1}\right\} \end{array}$$

The variation pattern observed in different color curves in Figure 2 indicates that the SIoU value exhibits correlation between the same target and different predicted fields of view bounding box. Moreover, it demonstrates distinguishability for targets of the same category but different scales. Among the four consecutive SIoU values obtained, those corresponding to small-scale bounding box targets exhibit relatively small values and display a decreasing trend. In contrast, the SIoU values for medium-scale bounding box targets are relatively larger, with an initial increase followed by a subsequent decrease. For large-scale bounding box targets, the SIoU values are relatively small and demonstrate an upward trend. These trends primarily emphasize the relative relationships among SIoU values, rather than the absolute values themselves.

**Figure 3:**
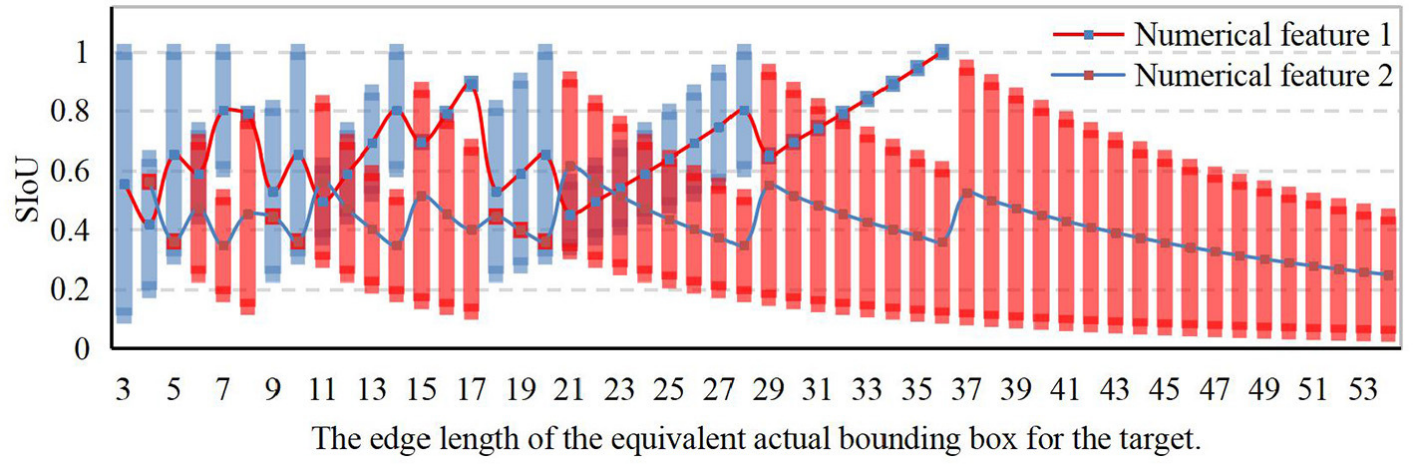
Statistical distribution of SIoU numerical ranges for all scale targets.

Figure 3 presents the statistical distribution of all corresponding SIoU values computed for equivalent target bounding box sizes ranging from 3 × 3 to 54 × 54. These calculations are performed when the observation view output feature planes of the three prediction branches are set to 56 × 56, 28 × 28, and 14 × 14, respectively. The SIoU values are categorized into two groups based on the size of the predicted view bounding box and the actual target bounding box. As depicted in the Figure 3, the SIoU numerical ranges for the majority of target exhibit considerable overlap and intersections with one another. This observation suggests that employing any volume of samples and training the model to extract the four required SIoU numerical features for targets of diverse scales indirectly enhances the extraction capability of relevant SIoU values for targets of other scales. Moreover, it indicates a weak dependence of the SIoU value on the scale statistical distribution of the dataset.

**Figure 4:**
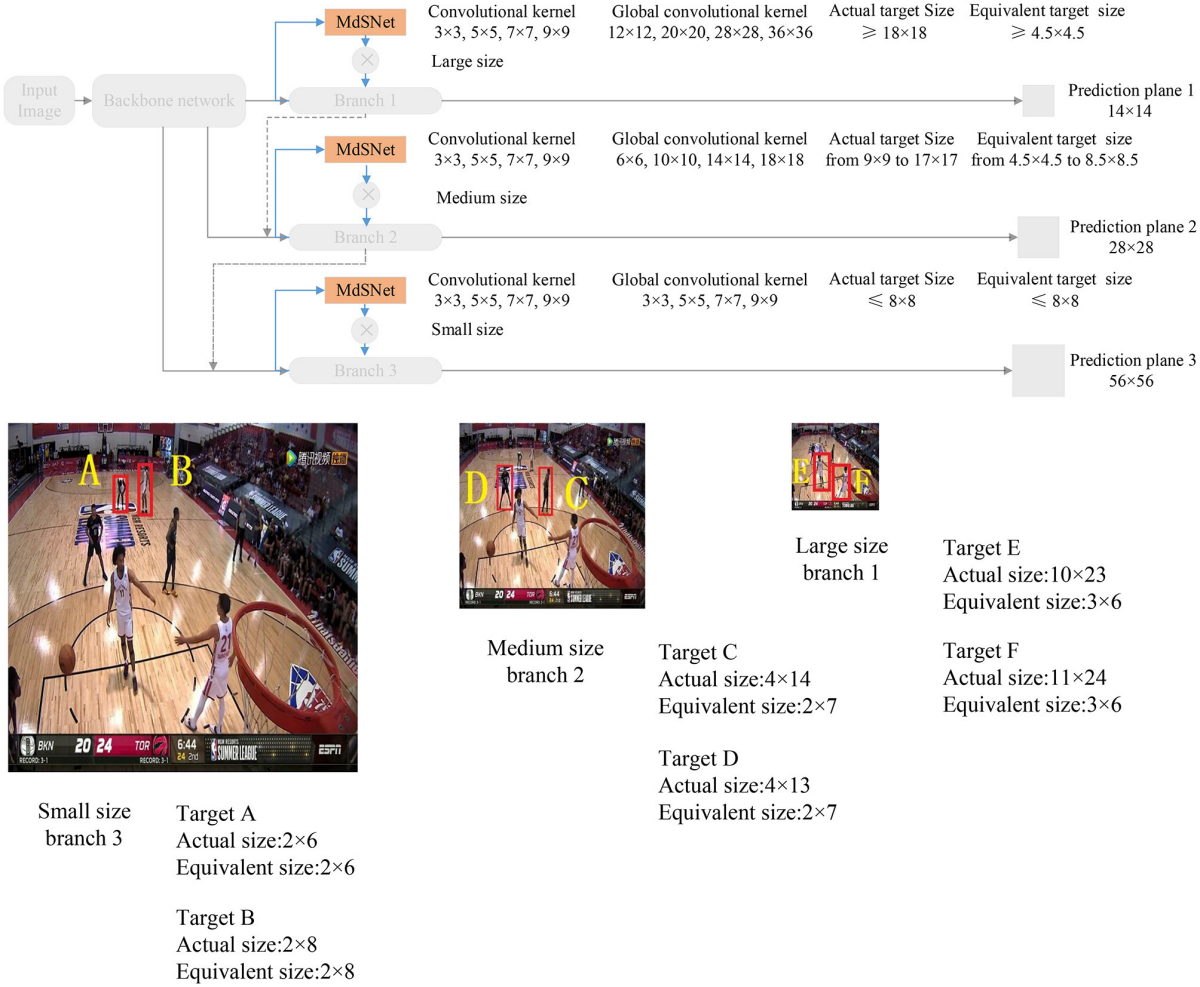
Guidelines for selecting target belonging branches.

When employing the SIoU value-based label to assist the depth Convolutional Neural Network in constructing a multi-scale attention plane, and under the condition where all branches share the same SIoU value, the network model can accommodate different scale targets through its multi-scale branch structure. Additionally, the predicted field of view bounding boxes at various scales can be efficiently replaced by globally equivalent predicted field of view bounding boxes in different branches, utilizing basic convolutional kernels with size of 3 × 3, 5 × 5, 7 × 7, and 9 × 9, respectively. As depicted in Figure 4, the input basketball game image comprises a total of 6 targets, consisting of 2 large-size targets, 2 medium-size targets, and 2 small-size targets. Following the aforementioned guidelines, the scale attention for targets A and B is assigned to the small-size branch 3, the scale attention for targets C and D is assigned to the medium-size branch 2, and the scale attention for targets E and F is allocated to the large-size branch 1.

## 3 Proposed method

Based on the SIoU label, we initially construct a network module to extract multi-dimensional distribution features. It utilizes coarse granularity scale attention formed by the explicit features of multiple scales to enhance multi-target detection with scale imbalance. Subsequently, leveraging the distinctive characteristic of a single target type in team sports, the K-medoids algorithm is enhanced by incorporating player bounding box information and statistical features, resulting in a fine-grained scale attention optimization algorithm. Finally, the proposed scale equalization algorithm is integrated with the SIoU label to jointly facilitate the training of the network model incorporating multi-scale attention.

### 3.1 Network module for SIoU feature extraction

**Figure 5:**
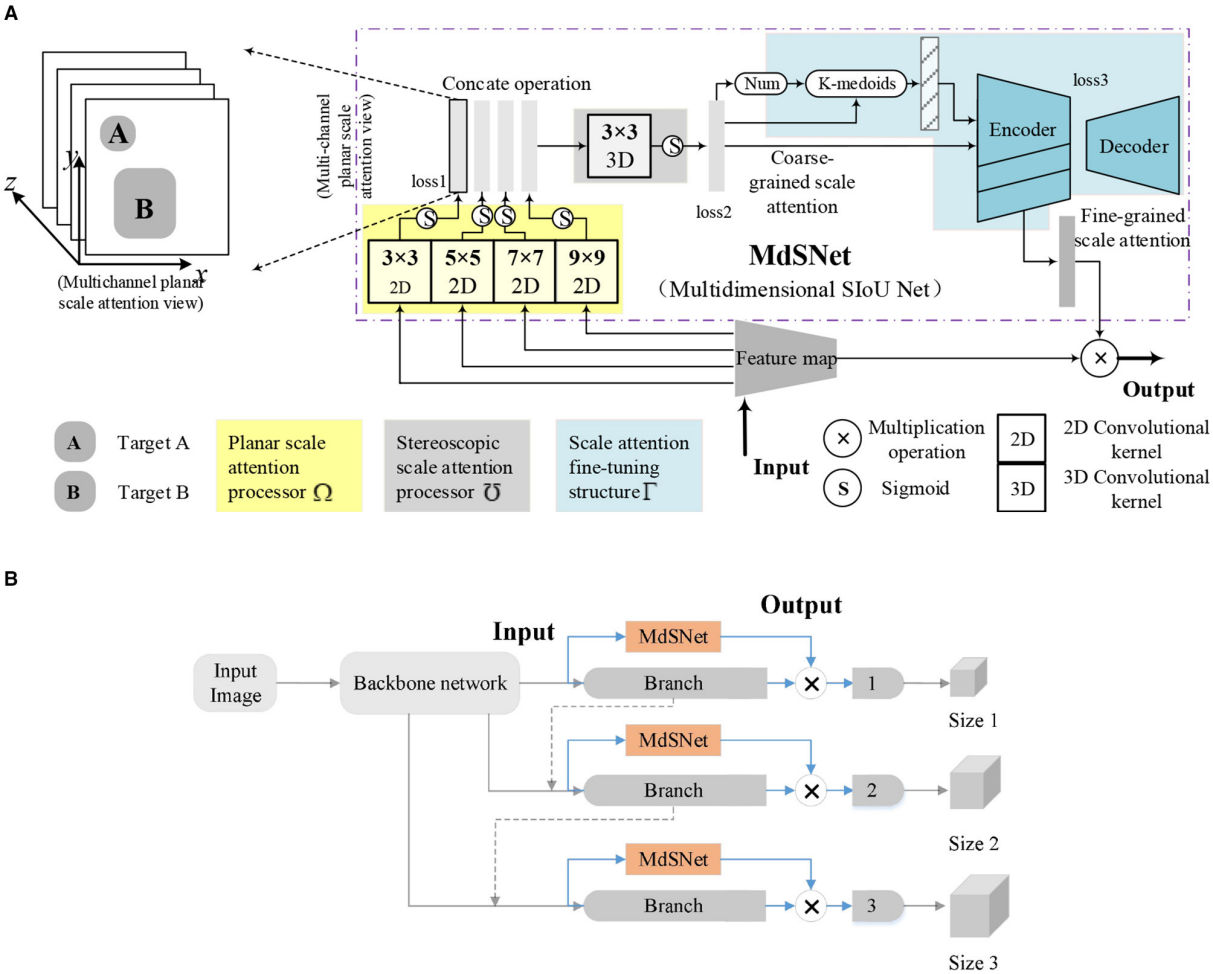
Schematic diagram of the MdSNet and its relationship with target detection model. **(A)** Schematic diagram of the MdSNet. **(B)** The relationship between MdSNet and a typical target detection model.

This article introduces a network module named MdSNet (Multidimensional SIoU Net) designed to extract multi-dimensional SIoU features generated by multi-scale targets through the application of multi-scale convolution kernels. As depicted in Figure 5A, MdSNet comprises three main components: a planar scale attention processor, a stereoscopic scale attention processor, and a scale attention fine-tuning structure. Their corresponding training loss functions are denoted as loss1, loss2, and loss3, respectively. Simultaneously, we illustrate the relationship between the MdSNet module and traditional object detection and localization models in Figure 5B. Ultimately, the module outputs a fine-grained scale attention feature plane.

The planar scale attention processor incorporates multi-scale convolutional kernels and sigmoid functions. The four sizes of convolutional kernels generate four planar scale attention feature maps for all corresponding targets in their respective scale branches. The resulting feature maps are then concatenated to form a multi-channel structure. The stereoscopic scale attention processor is composed of a 3D convolutional kernel and sigmoid functions. It takes multi-channel planar scale attention concatenation maps as input, producing coarse-grained scale attention planes, and predicting the number of potential targets within the planes. The scale attention fine-tuning structure comprises a statistical feature extraction process and a codec, ultimately yielding a fine-grained scale attention plane.

### 3.2 Process for coarse-grained attention generation

**Figure 6:**
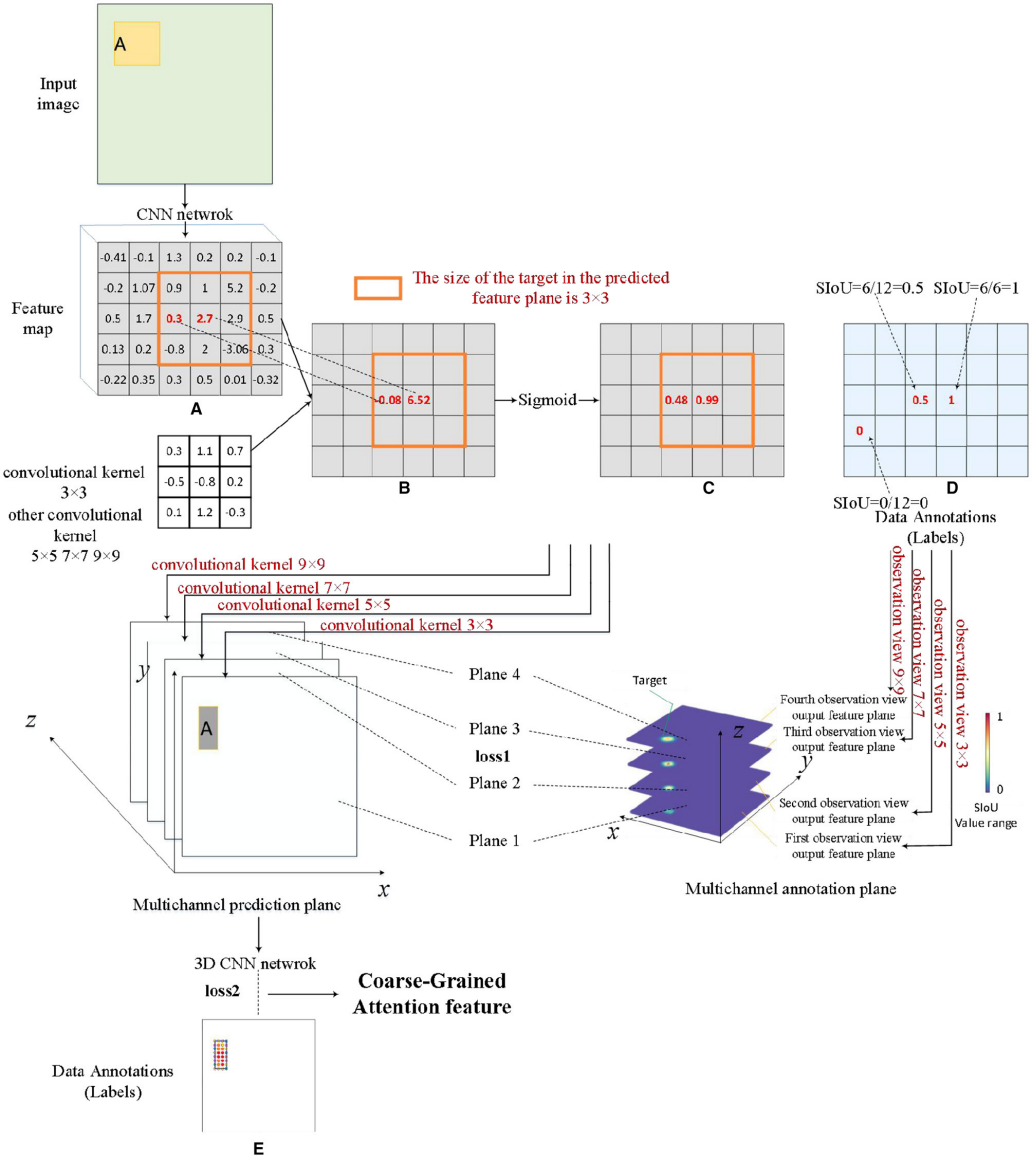
Coarse-grained attention feature generation process. **(A)** Feature map, **(B)** single channel feature map, **(C)** feature map after sigmoid function, **(D)** the intermediate process of data annotations operation, and **(E)** data annotations.

The planar scale attention processor and the stereoscopic scale attention processor collectively constitute the pivotal components of the SIoU multi-dimensional distribution feature extraction network module. The training process commences sequentially, considering both the sample volume of the dataset and the structure of the network model. Firstly, the planar scale attention processor is trained, and the data labels during training are generated based on equation (2). Figure 6A is a conventional feature map, while Figure 6B is a single channel feature map obtained using a fixed size convolution kernel. Figures 6C, D illustrate the predicted data and label data, respectively. At this stage, the loss function loss1 is constructed based on the L2 norm, which is the mean square error function, and the optimizer used is the Stochastic Gradient Descent (SGD) algorithm. The main objective of this training process is to discriminate the various SIoU numerical information generated by different scale bounding box targets under the influence of the same size convolutional kernel. The emphasis lies in obtaining the absolute distribution of SIoU features in the plane space, as expressed by each output channel feature map. Secondly, the stereoscopic scale attention processor is trained to improve the capability of extracting multi-dimensional SIoU features, with a particular emphasis on capturing the relative relationships between the SIoU values of each channel within the input multi-channel planar scale attention feature map. Data labels required for training are generated using a normal distribution, where the statistical distribution of each target is set as a normal distribution with parameter (μ, σ), serving to approximate the coarse-grained scale feature range. The specific values of this parameter can be determined through experimental evaluations. This is shown in Figure 6E. At this stage, the loss function loss2 is constructed based on the L2 norm.

### 3.3 Process for fine-grained attention generation

Scale attention fine-tuning structure employs real data to compensate for the subjectivity of the SIoU label in this study, and it aims to optimize the coarse-grained scale attention features produced by the MdSNet network module. This structure executes Algorithm 1, initially employing the enhanced K-medoids algorithm in conjunction with the number of targets predicted by the previous processor in the feature map to compute the center position of each target on the coarse-grained scale attention feature plane. Subsequently, the orientations of all targets are sorted using the Manhattan distance. Finally, through training with a codec and statistical interval estimation method, the confidence interval derived from real data guides the module to generate the best-matched confidence interval, achieving fine-tuning of scale attention.

**Algorithm 1:** Fine-grained scale attention optimization algorithm.

**Input**: Θ, coarse-grained scale feature plane; ℕ, the predicted number of targets in the feature map; Scale adjustment encoder quantity K and corresponding branch target basic size *w*_Anchor_.
**Output**: Fine-grained scale feature plane.	1: Center of target on Θ :	*C*_*p*_ = [(*cr*__*x*_1_, *cr*_*y*_1_), ⋯ , (*cr*_*x*_ℕ_, *cr*_*y*_ℕ_)]1 × ℕ_ ← Improved K-medoids and ℕ.	2: Use Manhattan distance function *f*_mhd_(▪) to sort the orientation of the target:	[(*cp*__*x*_1_, *cp*_*y*_1_), ⋯ , (*cp*_*x*_ℕ_, *cp*_*y*_ℕ_)]1 × ℕ_ = *f*_mhd_(*C*_*p*_),	*C*_*p*_ = [(*cr*__*x*_1_, *cr*_*y*_1_), ⋯ , (*cr*_*x*_ℕ_, *cr*_*y*_ℕ_)]1 × ℕ_, *C*_*p*_ predicted target.	[(*ct*__*x*_1_, *ct*_*y*_1_), ⋯ , (*ct*_*x*_ℕ_, *ct*_*y*_ℕ_)]1 × ℕ_ = *f*_mhd_(*C*_*t*_),	*C*_*t*_ = [(*ct*__*x*_1_, *ct*_*y*_1_), ⋯ , (*ct*_*x*_ℕ_, *ct*_*y*_ℕ_)]1 × ℕ_, *C*_*t*_ real target.	3: **for** λ ∈ [1, ℕ] **do**	Regional sample mean ${\bar{A}}_{r}^{\lambda }$, Regional sample variance related variable ${\bar{B}}_{r}^{\lambda }$	4: **for** κ ∈ [0, *K*] **do**	5: *p*_κ_ = *w*_Anchor_ ± κ * Δ, perform κ times of scaling.	6: Predict regional sample mean ${\bar{A}}_{p_{\kappa }}^{\lambda }$.	7: Predict regional sample variance related variable ${\bar{B}}_{p_{\kappa }}^{\lambda }$.	8: ${\bar{B}}_{p}^{\lambda }\leftarrow \min\left(\overset{\lambda }{\underset{p}{\bar{B}}},{\bar{B}}_{p_{\kappa }}^{\lambda }\right)$. smallest region sample variance related variable.	9: **end for**	10: loss←loss+loss_λ_, $\text{los}{\text{s}}_{\lambda }\leftarrow \text{loss}=\sqrt{{\left(\bar{A_{r}}-\bar{A_{p}}\right)}^{2}}+\sqrt{{\left(\bar{B_{r}}-\bar{B_{p}}\right)}^{2}}$.	11: **end for**

The essence of the K-medoids algorithm improvement resides in the distance calculation method between the associated feature points, as illustrated in equation (7). φ and τ are obtained based on equations (8) and (9), respectively, where (*x*_0_, *x*_1_)(*y*_0_, *y*_1_) represents the coordinate information of the two points, and *f*_size_ denotes the size of the current feature plane. Considering that competitive game images are resized to a standard size of 448 × 448 before being fed into the network model, the bounding boxes of players exhibit evident aspect ratio characteristics. Consequently, for distance calculation, an ellipse with a major-to-minor axes ratio of τ is constructed, and the ratio τ is adjusted based on the statistical distribution characteristics of the player's bounding box width and height.


(7) $$\begin{array}{l} L_{xy}={\left(\frac{x_{0}^{2}}{\varphi }+\frac{x_{1}^{2}}{\tau \cdot \varphi }-\frac{y_{0}^{2}}{\varphi }-\frac{y_{1}^{2}}{\tau \cdot \varphi }\right)}^{2} \end{array}$$

(8) $$\begin{array}{l} \varphi ={\left(x_{0}+\tan\left(\frac{1}{f_{\text{size}}}\right)\cdot x_{1}\right)}^{2} \end{array}$$

(9) $$\begin{array}{l} \tau =\frac{x_{1}^{2}}{\varphi -x_{0}^{2}} \end{array}$$

**Figure 7:**
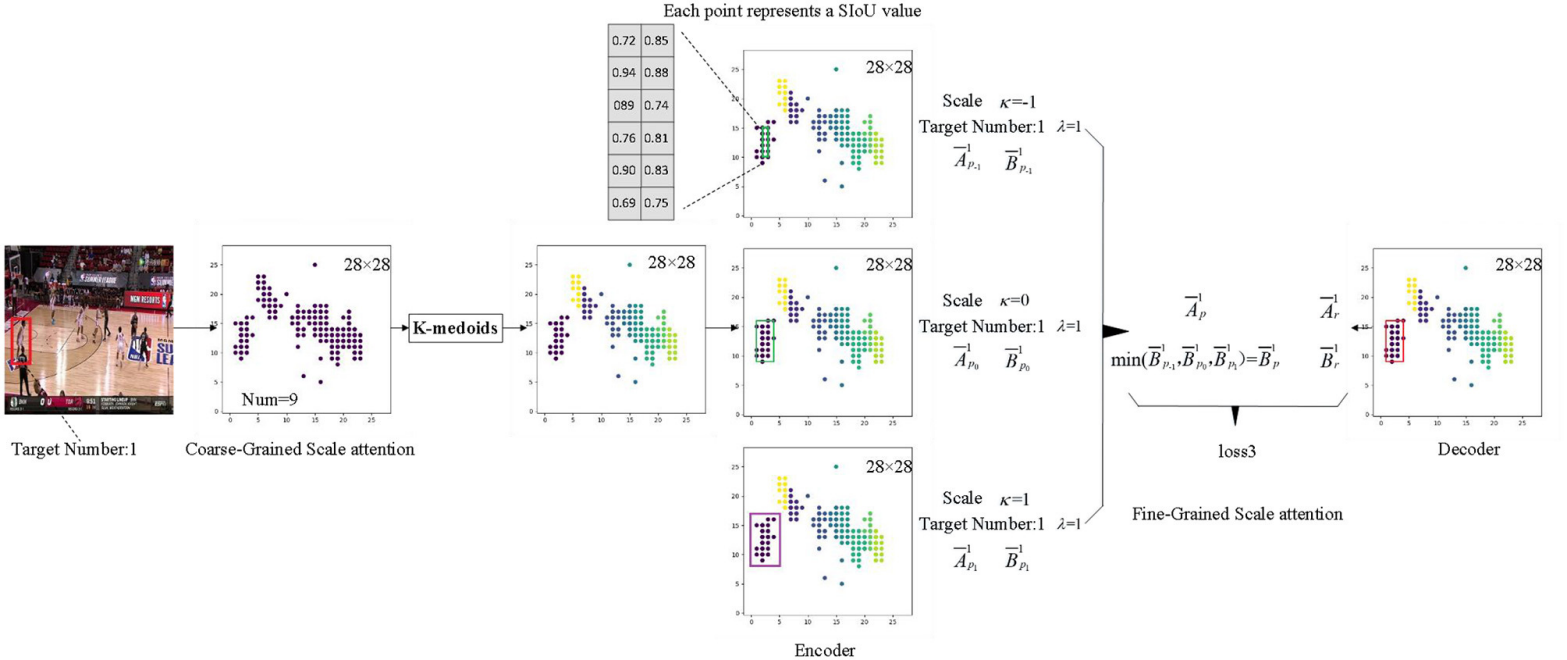
The specific generation process of fine-grained attention features.

The specific process is depicted in Figure 7. When implementing a codec, the confidence interval within the corresponding bounding box range serves as both the decoder and encoder. The confidence region range is determined using equation (10), where $\bar{A}$ represents the sample mean and $\bar{B}$ represents the interval width. $\bar{B}$ can be computed using equation (11), where $\bar{S}$ represents the square root of the sample variance, and *n* is the number of sample points. For coarse-grained scale attention planes, once the bounding box information for each target is established, it can be assumed that its scale features follow a normal distribution. Although the true mean and variance of the corresponding statistical distribution are unknown, confidence data within a certain bounding box can be used as a sampling sample to calculate its sample mean $\bar{A}$ and sample variance $\bar{S}$. Consequently, the confidence interval for the statistical mean μ at a confidence level 1-α can be computed. The decoder obtains the necessary bounding box information from the real data labels of the target. Given the fixed scale size of each branch adapted by the MdSNet network module, the encoder acquires the boundary box information from the boundary boxes obtained after multiple length and width expansions or contractions of each scale branch. By utilizing the feature information from the encoder with the narrowest confidence interval range (i.e., the encoder feature with the most concentrated scale feature data), along with the real label information from the decoder, the loss function is solved in accordance with equation (12), this corresponds to the loss3 in the figure.


(10) $$\begin{array}{l} \left(\bar{A}-\bar{B},\bar{A}+\bar{B}\right) \end{array}$$

(11) $$\begin{array}{l} \bar{B}=\frac{\bar{S}}{\sqrt{n}}t_{\alpha /2}\left(n-1\right) \end{array}$$

(12) $$\begin{array}{l} \text{loss}=\sqrt{{\left(\bar{A_{r}}-\bar{A_{p}}\right)}^{2}}+\sqrt{{\left(\bar{B_{r}}-\bar{B_{p}}\right)}^{2}} \end{array}$$

### 3.4 Scale equalization algorithm

The scale equalization algorithm equalizes image scale statistics that approximate a normal distribution. It achieves this by indirectly using the scaling factor $\gamma_{h,w}^{i,j}$, without directly altering the sample bounding box size in the dataset. The algorithm's purpose is to reduce missed detections of relatively small-scale bounding box targets within the dataset. Drawing inspiration from the image grayscale value equalization algorithm (Acharya and Kumar, 2021), we transform the probability density functions of the image height statistic *h* and width statistic *w*, following equations (13) and (14) respectively, to derive new statistics ϕ and ψ. Since *h* and *w* are independent of each other, ϕ and ψ are also independent, as indicated by their joint probability density as shown in equation (15).


(13) $$\begin{array}{l} \phi :H\left(h\right)=\int_{0}^{h}f\left(h\right)dh \end{array}$$

(14) $$\begin{array}{l} \psi :H\left(w\right)=\int_{0}^{w}f\left(w\right)dw \end{array}$$

(15) $$\begin{array}{l} f\left(w,h\right)=f\left(\phi \right)\cdot f\left(\psi \right) \end{array}$$

Since both ϕ and ψ follow a uniform distribution after transformation, *f*(*w, h*) = 1 also adheres to a uniform distribution probability density on 0 ≤ *w* ≤ 1 and 0 ≤ *h* ≤ 1. As a result, the statistical information of the non-balanced scale quantity in the dataset can be effectively balanced. The scaling factor $\gamma_{h,w}^{i,j}$, obtained through the equalization algorithm, and the SIoU label designed in this paper can be multiplied and fused following equation (16). The parameters $m_{h}^{i}$ and $n_{h}^{i}$ represents the quantity values of the *i*-th level of height statistics for targets in the source dataset before and after the execution of the algorithm, respectively, while $m_{w}^{j}$ and $n_{w}^{j}$ represent the quantity values of the *j*-th level of width statistic for targets in the source dataset before and after algorithm execution. The fundamental principle of this scale equalization lies in the utilization of scaling factors to introduce perturbations during the training process, particularly for targets with a large volume of specific scales, with the aim of mitigating overfitting.


(16) $$\begin{array}{l} \gamma_{h,w}^{i,j}=\frac{n_{h}^{i}}{m_{h}^{i}}\cdot \frac{n_{w}^{j}}{m_{w}^{j}} \end{array}$$

## 4 Experiment

### 4.1 Dataset and statistical distribution analysis

**Figure 8:**
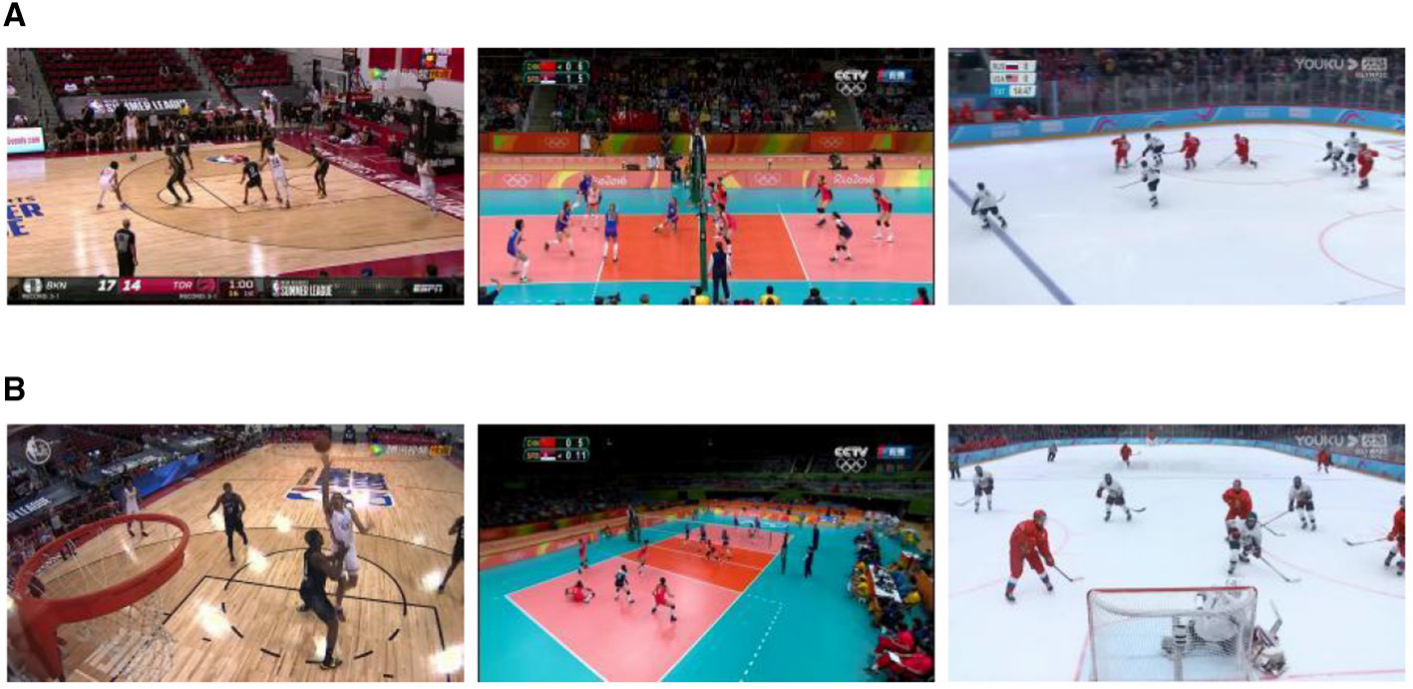
Sample images from the dataset. **(A)** Primary scale samples. **(B)** A limited number of small-scale and large-scale samples.

Current competitive game datasets predominantly encompass medium-scale bounding box samples, as depicted in Figure 8A, for player detection, often overlooking the relatively scarce instances of both small-scale and large-scale bounding box samples, illustrated in Figure 8B.

**Figure 9:**
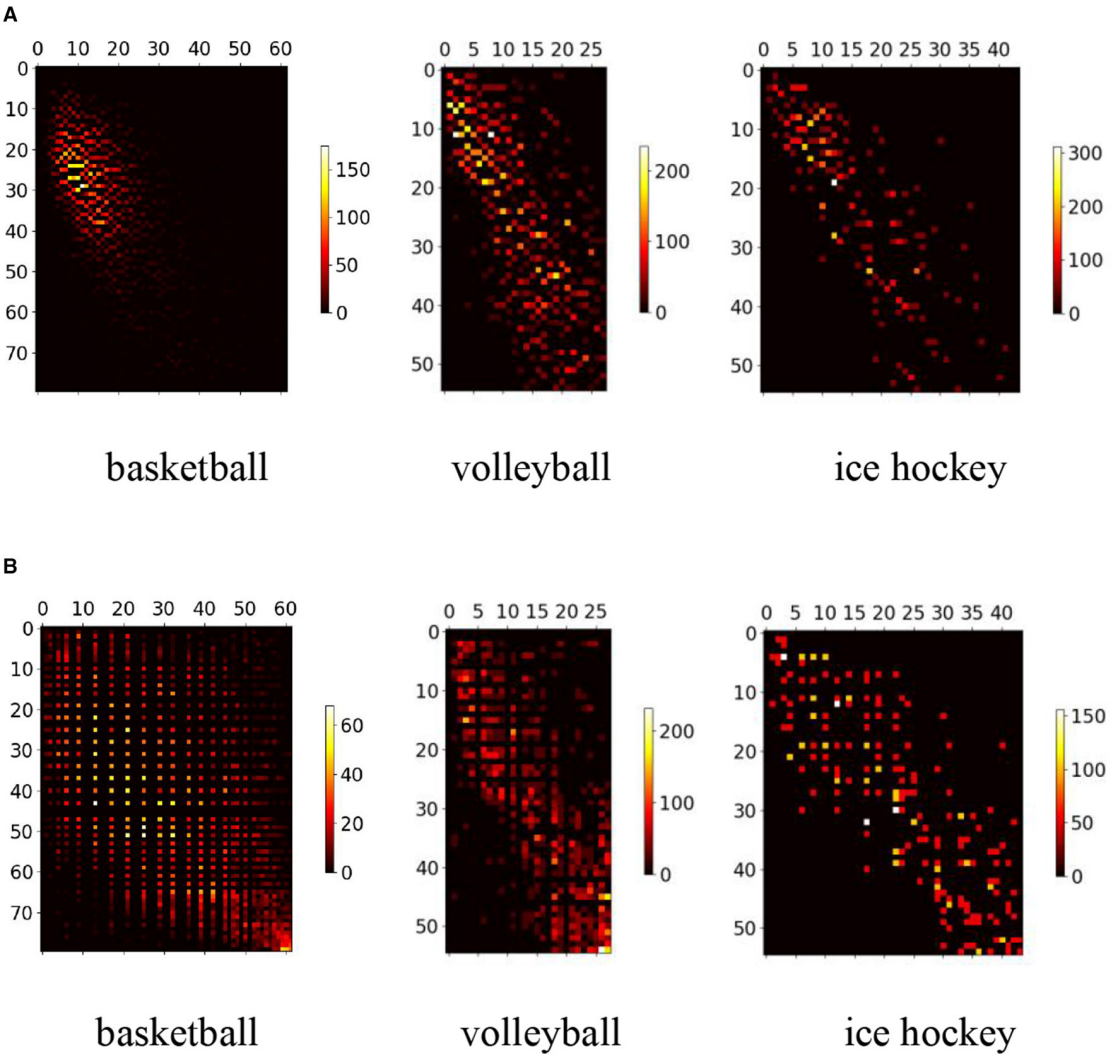
The scale distribution of the dataset. **(A)** The scale distribution of the original dataset. **(B)** The scale distribution after dataset scale equalization.

We undertake the reconstruction of a comprehensive competitive competition dataset that encompasses targets of diverse scale bounding boxes. Sample scale equalization, based on Algorithm 2, is then implemented. The dataset comprises three distinct game scenarios: basketball, volleyball, and ice hockey. Each scenario encompasses ~25 min of valid video sequences, each with a frame rate of 25. Extracting 5% of the image frames from the video, player information is annotated, resulting in around 15K, 13K, and 16K labels for basketball, volleyball, and ice hockey, respectively. The initial scale distribution of the dataset, depicted in Figure 9A, exhibits unevenness and approximately follows a normal distribution. Post-processing with Algorithm 2 yields the scale distribution depicted in Figure 9B, markedly enhancing overall distribution balance compared to the original dataset.

**Algorithm 2:** Sample Scale Equalization Algorithm.

**Input**: Height and Width of the bounding boxes of all samples and their quantities.	**Output**: Scaling factor $\gamma_{h,w}^{i,j}$.	1: *H, W* ← Grade height and width at certain intervals respectively.	2: *m*_*h*_, *m*_*w*_ ← *H*.size(), *W*.size(), Count the number of lengths and widths.	3: $H\left(h_{\xi }\right)=\sum_{\chi =0}^{\varsigma }f\left(h_{\chi }\right)\leftarrow :H\left(w\right)=\int_{0}^{w}f\left(w\right)dw.$	4: $\phi_{\varsigma }=H\left(h_{\varsigma }\right)=\sum_{\chi =0}^{\varsigma }f\left(h_{\chi }\right)=\sum_{\chi =0}^{\varsigma }m_{h}^{\chi }/m_{h}$, Length grade that exists after transformation.	5: $H\left(w_{\chi }\right)=\sum_{\varepsilon =0}^{\xi }f\left(h_{\varepsilon }\right)\leftarrow f\left(w,h\right)=f\left(\phi \right)\cdot f\left(\psi \right)$	6: $\psi_{{\;}_{\xi }}=H\left(w_{\xi }\right)=\sum_{\varepsilon =0}^{\xi }f\left(w_{\varepsilon }\right)=\sum_{\varepsilon =0}^{\xi }m_{w}^{\varepsilon }/m_{w}$, Widths grade that exists after transformation.	7: Restores ϕ_ς_ and ψ_ξ_ to the standard normalized grade value.	8: **for** *i* ∈ [1, *m*_*h*_], *i* ∈ [1, *m*_*w*_] **do**	9: $m_{h}^{i}\leftarrow H\left[i\right],m_{w}^{j}\leftarrow W\left[j\right]$, The quantity of each grade before transformation.	10: $n_{h}^{i}\leftarrow f_{\text{hisEqu}}\left(\phi_{\varsigma },m_{h},m_{h}^{i}\right),n_{w}^{j}\leftarrow f_{\text{hisEqu}}\left(\psi_{\xi },m_{w},m_{w}^{j}\right)$, Number of length and width grade after scale equalization, *f*_hisEqu_(▪) histogram equalization algorithm.	11: $\gamma_{h,w}^{i,j}=\frac{n_{h}^{i}}{m_{h}^{i}}\cdot \frac{n_{w}^{j}}{m_{w}^{j}}$, Scaling factor.	12: **end for**

### 4.2 Experiment on multi-scale attention generation

**Figure 10:**
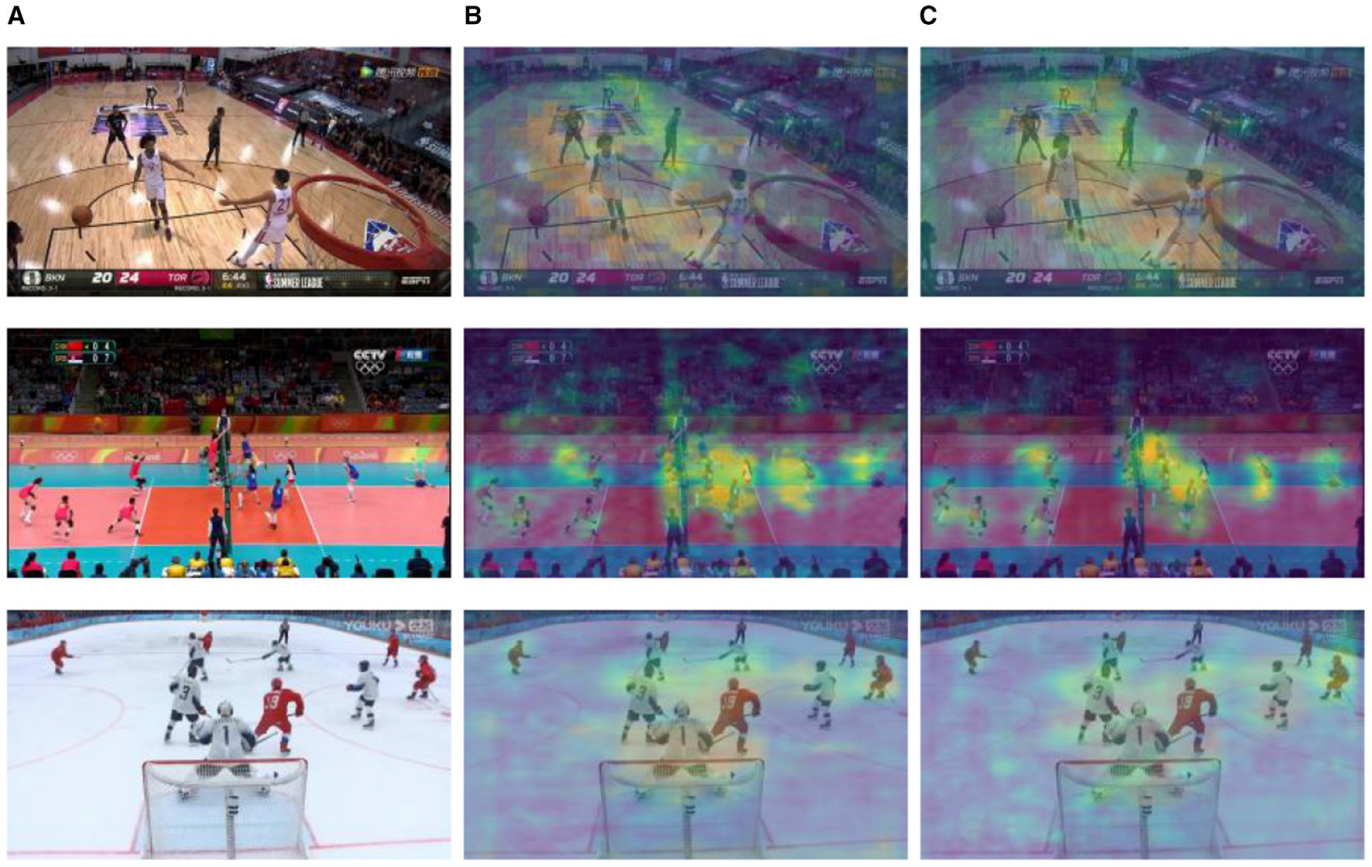
Comparison between coarse-grained and fine-grained scales attention. **(A)** Original image **(B)** Coarse-grained scale attention **(C)** Fine-grained scale attention.

The process of formulating scale attention predominantly encompasses acquiring two categories of information: the coarse-grained features of multi-scale attention and the fine-grained features of multi-scale attention. In the experiment, the ResNet architecture was adopted as the backbone network, leading to the construction of three scale attention branches: large, medium, and small. The ultimate dimensions of the predicted feature planes were 56 × 56, 28 × 28, and 14 × 14, respectively. To acquire coarse-grained information of multi-scale attention features, the hyperparameters were set as follows: μ = 0.85 and σ = 0.15, utilized during the generation of training labels. For the fine-grained information of multi-scale attention features, following the principles outlined in Algorithm 1, corresponding quantity fine-tuning encoders were designed for the three scale branches. The visualization outputs of the experience are depicted in Figure 10, where Figure 10A is the original image. These results illustrate that coarse-grained scale attention, Figure 10B, effectively segregates the scale features of the target for detection and enhances its positional information. Additionally, fine-grained scale attention, Figure 10C, further refines the precision and concentration of potential target positions, building upon the foundation laid by coarse-grained scale attention. Certainly, fine-grained scale attention not only enhances detection accuracy but also results in a several-fold increase in the overall runtime of the network model. This is especially due to the improved K-medoids algorithm, which adds considerable time overhead. Therefore, the scale attention model is better suited for offline video processing, similar to the one investigated in this article.

### 4.3 Comprehensive experiment

This section presents three comprehensive sets of experiments concerning multi-scale player detection. The first set is ablation experiments focusing on the three fundamental processes outlined in our method, aiming to evaluate the efficacy of each process. The second set involves experiments conducted with a dataset volume of approximately 10%, serving as a preliminary validation of the proposed method's capacity to enhance target detection accuracy. In the third set of experiments, algorithmic comparisons are conducted across various dataset volumes, serving to underscore the limited influence of sample size distribution on the multi-scale attention model.

#### 4.3.1 Ablation experiment

**Figure 11:**
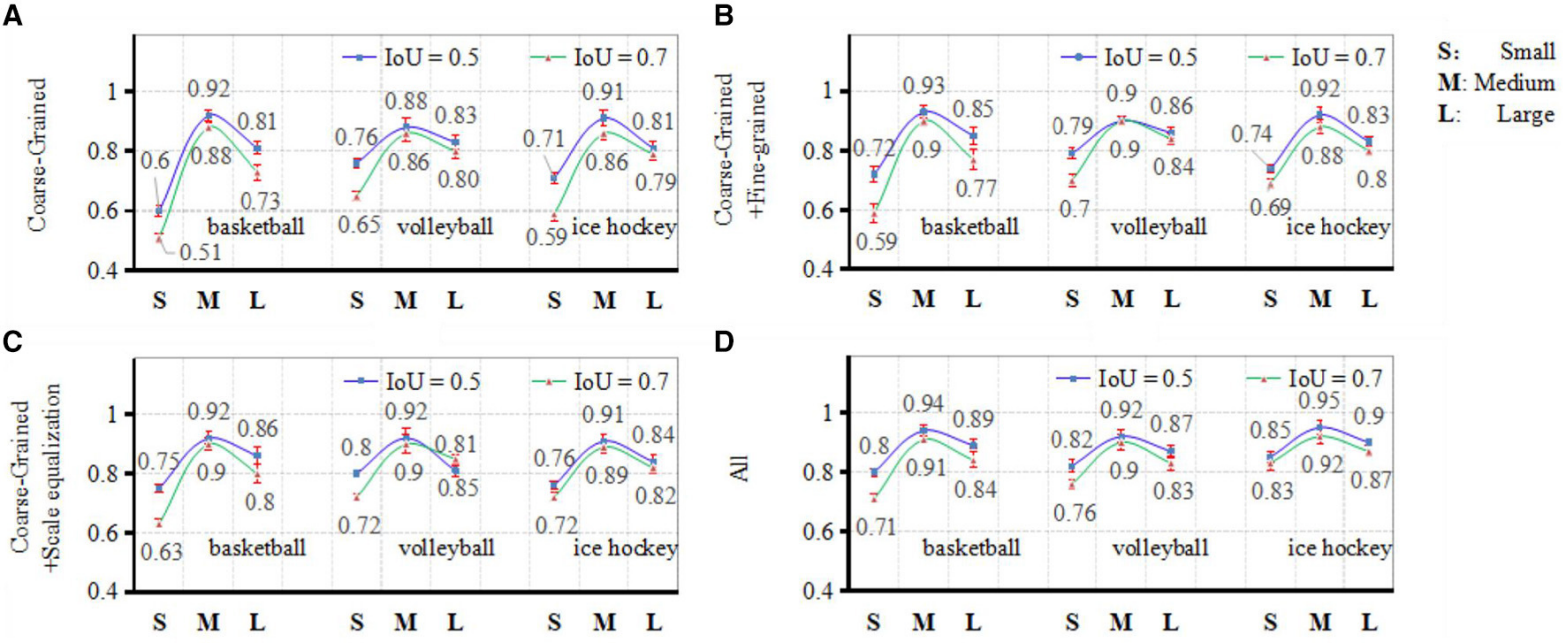
Results from ablation experiments comparison. **(A)** Coarse-grained, **(B)** coarse-grained and fine-grained, **(C)** coarse-grained and scale equalization, and **(D)** all.

The experimental findings, presented in Figure 11, depict ablation experiments conducted on the three core processes encompassing coarse-grained scale attention, fine-grained scale attention, and scale equalization, as formulated in the methodology of this article.

The evaluation metrics employed in this experiment are computed according to equation (17), where TP denotes the count of correctly predicted positive player instances, FP signifies the count of erroneously predicted positive player instances, and FN represents the count of erroneously predicted negative player instances. In the course of the experiment, the IoU thresholds for player detection were set at 0.5 and 0.7, respectively. The accuracy of target detection was assessed across four scenarios: solely employing coarse-grained scale attention, utilizing both coarse-grained and fine-grained scale attention, incorporating coarse-grained scale attention and the scale equalization algorithm, and integrating all three core processes. Analyzing the results reveals that coarse-grained scale attention serves as the fundamental framework for achieving multi-scale object detection in ball games. Fine-grained attention functions as a secondary refinement of coarse-grained attention, showcasing more pronounced enhancements in detection outcomes particularly under higher IoU requirements. The scale equalization algorithm is particularly effective in enhancing the detection capability for maximum and minimum scale bounding box targets within smaller sample volume, yielding notably improved effects compared to fine-grained scale attention.


(17) $$\begin{array}{l} \text{ACC=}\frac{\text{TP}}{\text{TP+FP+FN}} \end{array}$$

#### 4.3.2 Algorithm comparison experiment under low data volume

**Figure 12:**
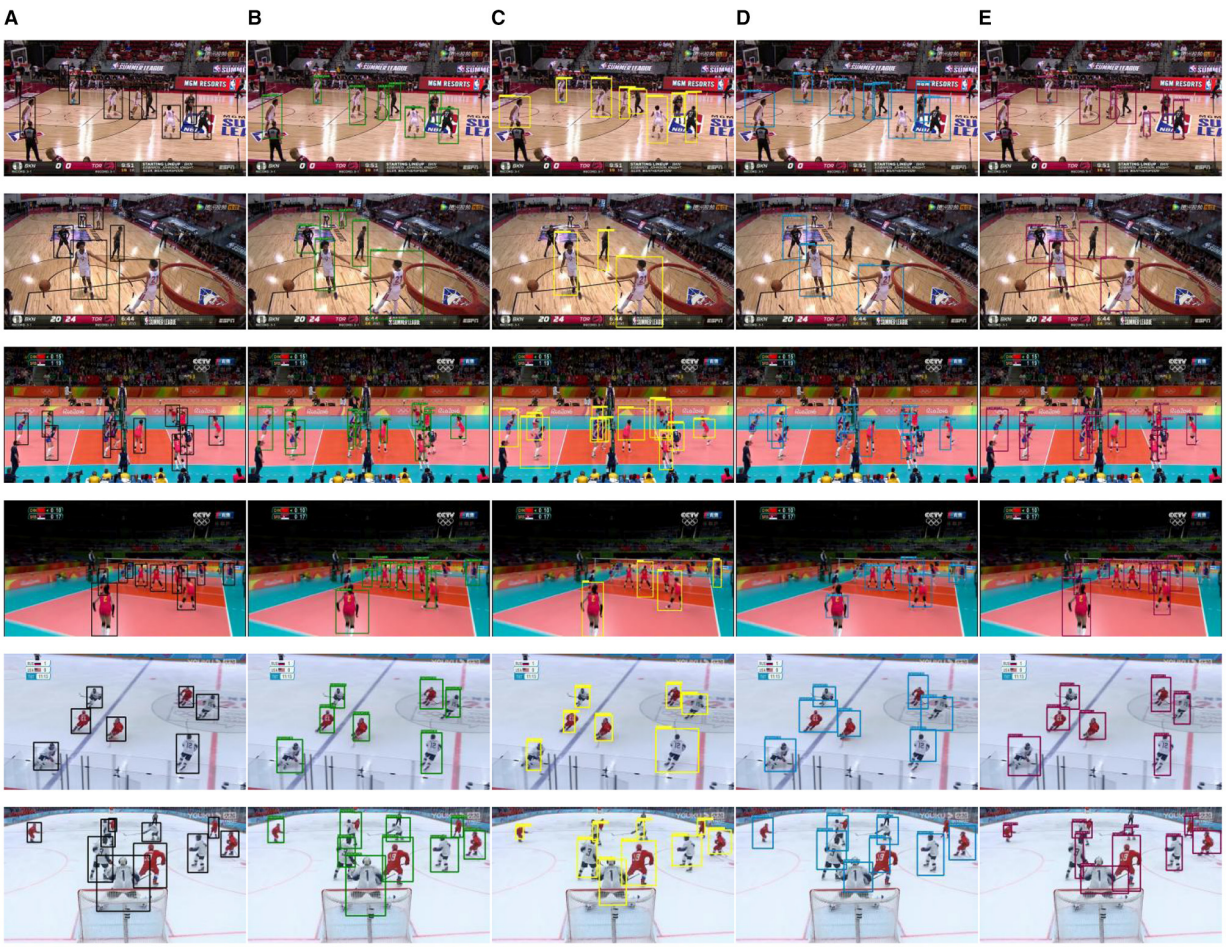
Results of player detection comparison. **(A)** Data labels. **(B)** Ours. **(C)** FPN. **(D)** PANet. **(E)** BiFPN.

To provide an initial validation of the capability of multi-scale attention to enhance the accuracy of conventional object detection algorithms, a subset amounting to approximately 10% of the player detection dataset was extracted. Leveraging the YOLOv3 algorithm and pretraining the backbone network on the PETA dataset, comparative experimental results were obtained for the approach presented in this article, the approach augmented with the FPN (Zhao et al., 2019) module, the approach augmented with the PANet (Bochkovskiy et al., 2020) module, and the approach augmented with the BiFPN (Zhang et al., 2021) module. As illustrated in Figure 12, the images in the odd-numbered rows depict the detection results of players enclosed within medium-scale bounding boxes. Conversely, the images in the even-numbered rows encompass the detection outcomes of players enclosed by bounding boxes of maximum or minimum scale.

Analysis reveals that the algorithm proposed by us demonstrates superior detection accuracy for a limited volume subset of extremely small-scale bounding box targets. Moreover, for a relatively small volume subset of extremely large-scale bounding box targets, the IoU indices of targets detected by this algorithm are notably improved. Across the dataset, all algorithms exhibit comparable detection capabilities for medium-scale bounding box targets. The quantitative comparison results for these observations are tabulated in Table 1.

**Table 1:** Comparison of player detection normalization results for algorithms with a 10% data volume.

**Algorithm**	**Data volume proportion**	**Basketball**			**Volleyball**			**Ice hockey**		
		**S**	**M**	**L**	**S**	**M**	**L**	**S**	**M**	**L**
YOLOv3+FPN		0.33	0.82	0.48	0.29	0.81	0.43	0.39	0.89	0.51
YOLOv3+PANet		0.38	**0.87**	**0.51**	0.31	0.79	**0.46**	0.41	0.90	0.58
YOLOv3+BiFPN		0.34	0.84	0.47	0.32	**0.82**	0.41	0.40	0.87	**0.60**
RetinaNet+AMF	10%	0.37	0.81	0.44	0.31	0.80	0.44	0.41	0.91	**0.60**
DeepPlayer		0.42	0.84	**0.51**	0.37	0.80	**0.48**	0.47	**0.91**	**0.61**
YOLOVX+ESPHead		**0.44**	0.85	**0.50**	0.32	**0.82**	**0.46**	**0.51**	0.90	0.55
YOLOv6		0.43	**0.88**	0.48	**0.40**	**0.81**	0.45	0.49	**0.92**	0.53
YOLOv3+MdSNet(Ours)		**0.57**	0.86	0.47	**0.51**	0.80	**0.48**	**0.62**	**0.91**	0.56

Bold numbers represent optimal and suboptimal data, respectively.

#### 4.3.3 Comparative experiment of algorithms across varied data volumes

Four additional comparative algorithms were introduced (Lin et al., 2017; Zhang et al., 2020; Ge et al., 2021; Li et al., 2022). Subsequent to training on comprehensive basketball, volleyball, and ice hockey datasets, the accuracy of player detection was computed at an IoU threshold of 0.5. Ultimately, for players of both very small and very large scale bounding box within the dataset, the proposed method showcased improvements of 11%, 7%, 15%, 8%, 9%, and 4%, respectively, in comparison to the optimal method. The comprehensive experimental results are illustrated in Tables 1–4, encompassing the detection quantification outcomes obtained for approximately 10%, 30%, 50%, and the 100% volume datasets, respectively. By further considering the statistical distribution information in Figure 9, it becomes evident that with an equivalent volume of data, the model augmented with both scale attention and the scale equalization algorithm exhibits distinct advantages in the detection of players at the maximum and minimum scale bounding box. This distinction is particularly pronounced in the case of basketball player detection. This observation can be attributed to the relatively limited quantity of minimum and maximum scale bounding box targets present within the basketball player detection dataset, thereby leading to a more pronounced imbalance in scale distribution. Concurrently, it is discernible that with the expansion of dataset volume, the approach delineated by us consistently refines the detection precision for maximum and minimum scale bounding box targets. Nevertheless, the missed detection probability for the other seven algorithms showcases minimal reduction. This outcome is rooted in the possibility that the scale distribution within the sampled dataset may mirror that of the complete dataset. This observation underscores the pronounced reliance of these algorithms on the scale statistical distribution attributes intrinsic to the dataset. Regrettably, they may lack the capability to rectify inaccuracies stemming from scale imbalance. In contrast, the algorithm proposed by us evinces reduced sensitivity to dataset scale balance. It demonstrates a weaker interdependence on the dataset's scale distribution characteristics when compared to the other seven algorithms.

**Table 2:** Comparison of player detection normalization results for algorithms with a 30% data volume.

**Algorithm**	**Data volume proportion**	**Basketball**			**Volleyball**			**Ice hockey**		
		**S**	**M**	**L**	**S**	**M**	**L**	**S**	**M**	**L**
YOLOv3+FPN		0.36	0.89	0.52	0.30	0.87	0.46	0.44	0.91	0.60
YOLOv3+PANet		0.37	**0.91**	0.54	0.33	0.86	0.41	0.47	0.90	0.63
YOLOv3+BiFPN		0.40	0.88	0.49	0.36	**0.89**	0.49	0.43	0.93	0.61
RetinaNet+AMF	30%	0.46	0.90	0.54	0.37	**0.88**	0.52	0.50	**0.94**	0.63
DeepPlayer		**0.57**	**0.91**	0.68	0.48	0.86	**0.61**	0.59	0.92	0.64
YOLOVX+ESPHead		0.55	**0.91**	**0.70**	0.44	0.86	0.59	**0.62**	0.91	**0.69**
YOLOv6		**0.57**	0.89	0.62	**0.56**	**0.88**	**0.60**	0.61	**0.94**	0.65
YOLOv3+MdSNet(Ours)		**0.71**	0.87	**0.74**	**0.68**	0.84	**0.72**	**0.70**	0.92	**0.75**

Bold numbers represent optimal and suboptimal data, respectively.

**Table 3:** Comparison of player detection normalization results for algorithms with a 50% data volume.

**Algorithm**	**Data volume proportion**	**Basketball**			**Volleyball**			**Ice hockey**		
		**S**	**M**	**L**	**S**	**M**	**L**	**S**	**M**	**L**
YOLOv3+FPN		0.43	0.90	0.55	0.37	0.88	0.49	0.52	0.90	0.65
YOLOv3+PANet		0.41	**0.92**	0.54	0.35	**0.92**	0.47	0.55	**0.93**	0.60
YOLOv3+BiFPN		0.49	0.89	0.57	0.42	0.90	0.50	0.58	0.91	0.61
RetinaNet+AMF	50%	0.52	0.90	0.63	0.46	0.90	0.58	0.64	0.92	0.70
DeepPlayer		0.48	0.91	0.74	0.51	0.91	0.67	0.61	0.92	0.74
YOLOVX+ESPHead		0.61	0.91	**0.75**	0.54	0.91	0.69	0.68	0.92	**0.81**
YOLOv6		**0.65**	**0.92**	0.74	**0.63**	**0.93**	**0.72**	**0.73**	**0.93**	0.77
YOLOv3+MdSNet(Ours)		**0.75**	**0.93**	**0.81**	**0.70**	0.89	**0.79**	**0.78**	**0.94**	**0.84**

Bold numbers represent optimal and suboptimal data, respectively.

**Table 4:** Comparison of player detection normalization results for algorithms with a 100% data volume.

**Algorithm**	**Data volume proportion**	**Basketball**			**Volleyball**			**Ice hockey**		
		**S**	**M**	**L**	**S**	**M**	**L**	**S**	**M**	**L**
YOLOv3+FPN		0.48	0.93	0.58	0.41	0.90	0.53	0.65	0.93	0.62
YOLOv3+PANet		0.50	**0.95**	0.55	0.46	**0.93**	0.55	0.63	0.90	0.63
YOLOv3+BiFPN		0.49	0.90	0.60	0.42	0.91	0.51	0.65	**0.96**	0.67
RetinaNet+AMF	100%	0.55	0.93	0.67	0.49	**0.93**	0.62	0.68	**0.97**	0.73
DeepPlayer		0.51	0.92	**0.84**	0.44	**0.94**	0.76	0.72	**0.96**	0.77
YOLOVX+ESPHead		0.64	0.92	0.76	0.58	**0.94**	0.74	0.71	0.93	**0.86**
YOLOv6		**0.69**	0.93	**0.82**	**0.67**	0.92	**0.79**	**0.76**	0.95	0.81
YOLOv3+MdSNet(Ours)		**0.80**	**0.94**	**0.89**	**0.82**	0.92	**0.87**	**0.85**	0.95	**0.90**

Bold numbers represent optimal and suboptimal data, respectively.

## 5 Conclusion

This article initiates the concept of SIoU and meticulously scrutinizes its viability as a label for explicitly conveying multi-scale attributes. Subsequently, a network module is devised to extract the multi-dimensional distribution characteristics inherent to SIoU features, leveraging it to bolster the precision of multi-scale object detection within ball team sports. This module primarily encompasses a two-tiered granularity scale attention generation mechanism. The initial tier deploys an array of 2D convolutional kernels to derive numerous planar scale attentions, which are then merged with 3D convolutional kernels to construct spatial scale attention of 3D spatial features, culminating in the creation of a coarse-grained scale attention feature plane. The subsequent tier involves an enhanced K-medoids algorithm, coupled with interval estimation to establish a codec, thereby giving rise to a fine-grained scale attention feature plane. By harnessing label training models to ascertain the interrelated dynamics among SIoU numerical features during the extraction of attention features across multiple 2D plane scale levels and 3D spatial scale dimensions, the prominence of their absolute numerical attributes is diminished. Consequently, the process of scale attention generation becomes less predicated on the intricate scale distribution attributes within the dataset, thereby primarily mitigating the challenge of missed detections pertaining to targets of maximal and minimal scale bounding box. Furthermore, the integration of sample scale equalization algorithms into the model training procedure disrupts the overfitting tendency observed during training for specific scale bounding box targets with abundant instances. This augmentation further enhances the accuracy of multi-scale target detection, particularly for very small and very large scale bounding box players that appear less frequently. Building upon the findings of this current study, future research will place heightened emphasis on unraveling the interpretability and controllability of convolutional neural networks as a means of advancing the capabilities for multi-scale object detection.

## Data availability statement

The raw data supporting the conclusions of this article will be made available by the authors, without undue reservation.

## Author contributions

PZ: Writing – original draft. JL: Writing – review & editing.
